# The Radiogenomic Landscape of Clear Cell Renal Cell Carcinoma: Insights into Lipid Metabolism through Evaluation of ADFP Expression

**DOI:** 10.3390/diagnostics14151667

**Published:** 2024-08-01

**Authors:** Federico Greco, Andrea Panunzio, Caterina Bernetti, Alessandro Tafuri, Bruno Beomonte Zobel, Carlo Augusto Mallio

**Affiliations:** 1 Department of Radiology, Cittadella della Salute, Azienda Sanitaria Locale di Lecce, Piazza Filippo Bottazzi, 2, 73100 Lecce, Italy; 2 Research Unit of Radiology, Department of Medicine and Surgery, Università Campus Bio-Medico di Roma, Via Alvaro del Portillo, 21, 00128 Roma, Italy; c.bernetti@policlinicocampus.it (C.B.); b.zobel@policlinicocampus.it (B.B.Z.); c.mallio@policlinicocampus.it (C.A.M.); 3 Department of Urology, “Vito Fazzi” Hospital, Piazza Filippo Muratore, 1, 73100 Lecce, Italy; panunzioandrea@virgilio.it (A.P.); tafuri.alessandro@gmail.com (A.T.); 4 Fondazione Policlinico Universitario Campus Bio-Medico, Via Alvaro del Portillo, 200, 00128 Roma, Italy

**Keywords:** ADFP, adipose tissue, clear cell renal cell carcinoma, computed tomography, lipid metabolism, radiogenomics

## Abstract

This study aims to explore the relationship between radiological imaging and genomic characteristics in clear cell renal cell carcinoma (ccRCC), focusing on the expression of adipose differentiation-related protein (ADFP) detected through computed tomography (CT). The goal is to establish a radiogenomic lipid profile and understand its association with tumor characteristics. Data from The Cancer Genome Atlas (TCGA) and the Cancer Imaging Archive (TCIA) were utilized to correlate imaging features with adipose differentiation-related protein (ADFP) expression in ccRCC. CT scans assessed various tumor features, including size, composition, margin, necrosis, and growth pattern, alongside measurements of tumoral Hounsfield units (HU) and abdominal adipose tissue compartments. Statistical analyses compared demographics, clinical–pathological features, adipose tissue quantification, and tumoral HU between groups. Among 197 patients, 22.8% exhibited ADFP expression significantly associated with hydronephrosis. Low-grade ccRCC patients expressing ADFP had higher quantities of visceral and subcutaneous adipose tissue and lower tumoral HU values compared to their high-grade counterparts. Similar trends were observed in low-grade ccRCC patients without ADFP expression. ADFP expression in ccRCC correlates with specific imaging features such as hydronephrosis and altered adipose tissue distribution. Low-grade ccRCC patients with ADFP expression display a distinct lipid metabolic profile, emphasizing the relationship between radiological features, genomic expression, and tumor metabolism. These findings suggest potential for personalized diagnostic and therapeutic strategies targeting tumor lipid metabolism.

## 1. Introduction

The correlation of imaging features with genomics is called radiogenomics [1,2]. The macroscopic expression of molecular processes revealed with imaging constitutes radiologic phenotypes [1,2]. The Cancer Genome Atlas (TCGA) Research Network contains data on numerous clear cell renal cell carcinoma (ccRCC) gene expressions and mutations [3,4]. Radiogenomics offers a great number of advantages: radiogenomics is not invasive, like biopsy sampling, and is free from potential complications related to biopsy. The biopsy samples acquire data on the sample taken and not on the entire tumor; the radiogenomics of the entire tumor volume instead allows genomic data on the entire neoplasm to be obtained, giving the opportunity, in this way, to obtain data on the entire tumor’s heterogeneity. Acquiring whole-tumor genomic data is fundamental for the prognosis of the disease, as biopsy samples may not present gene expressions or gene mutations that are fundamental for planning the target therapy, which is necessary for the treatment of the disease. Radiogenomics allows for the evaluation of the “genomic status” over time during the patient’s follow-up, also evaluating genomic variations in relation to therapies. Thanks to radiogenomics, multiple lesions can be analyzed in parallel. Furthermore, imaging allows us to quantify body composition and associate it with genomic data, as was the case for ccRCC patients [5,6].

Adipose differentiation-related protein (ADFP) is one of the fundamental proteins for fatty acid uptake and for the formation and stabilization of lipid storage droplets [7,8]. ADFP is highly upregulated in ccRCC at both the transcriptional and protein levels [9]. High expression of ADFP is also present in adipocytes [10]. ADFP is a hypoxia-inducible gene, and its transcriptional activation is mediated by hypoxia-inducible factor (HIF) [11]. The von Hippel Lindau protein (VHLp) forms a complex with other proteins that determines the degradation of HIF; the VHL mutation determines the inactivation of VHL with consequent failure of HIF degradation, which determines the activation of pro-angiogenic pathways and cell growth [12,13,14,15]. This suggests that VHL mutation, through the lack of HIF inactivation, may determine up-regulation of ADFP in ccRCC [9].

To date, radiogenomics of ADFP expression in ccRCC patients have not been evaluated. The aim of this study is to investigate the CT signs of ADFP expression in ccRCC patients, also analyzing the abdominal adipose tissue distribution considering the activity of ADFP. We hypothesize that, using the CT approach, it is possible to delineate a radiogenomic lipid profile in ccRCC through the evaluation of ADFP expression.

## 2. Materials and Methods

### 2.1. The Cancer Genome Atlas

The Cancer Genome Atlas (TCGA), funded by the National Cancer Institute and the National Human Genome Research Institute (NHGRI), is an atlas of genetic changes in more than 20 types of cancer, including ccRCC. The tissue samples were sent by all participating institutions and, after receiving institutional review board approval, were subjected to complete multiplatform genomic characterization and analysis. TCGA consortium has created molecular profiles for over ten thousand samples from various cancer types, utilizing a range of data platforms such as DNA methylation, copy number variations, RNA expression, and protein expression [16].

The TCGA Kidney Renal Clear Cell Carcinoma (KIRC), part of the TCGA initiative, assessed gene expression using RNA sequencing analysis. The TCGA-KIRC data collection aims to foster a research community dedicated to linking cancer phenotypes with genotypes. This is achieved by providing clinical images corresponding to subjects from TCGA. While clinical, genetic, and pathological data are available in the Genomic Data Commons Data Portal, radiological data can be accessed through The Cancer Imaging Archive (TCIA). Tissues for TCGA were sourced globally to meet accrual targets. TCIA is a National Cancer Institute-supported anonymized image repository that is used to upload pretreatment medical images in DICOM format. TCIA organizes its data into “collections”, which generally consist of patient groups linked by a specific disease (e.g., lung cancer), imaging modality or type (like magnetic resonance imaging, CT, or digital histopathology), or research focus. When accessible, additional data supporting the images, such as patient outcomes, treatment information, genomics, and image analyses, are also included.

Imaging data from TCIA and tissue samples from the TCGA are linked by a unique identifier and are accessible for public download [17].

### 2.2. Imaging Features

The imaging features evaluated through the CT approach for each ccRCC are: size (in mm), composition (solid or cystic), margin (well-defined or ill-defined), necrosis (detected only for solid tumors: 0%, 1–33%, 34–66% or >66%), growth pattern (endophytic, <50% exophytic, or ≥50% exophytic), calcification (absent or present), laterality (left or right), presence or absence of collateral vascular supply (i.e., enlarged renal capsular veins macroscopically visible at CT or magnetic resonance imaging studies), intralesional hemorrhage, infiltration, collecting system invasion, hydronephrosis, renal artery thrombosis, and renal vein thrombosis [18,19,20]. Additional CT features are the quantity of abdominal adipose tissue (in cm^2^), Gerota’s fascia thickening (absent or present), perirenal fat stranding (absent or present) [5,6,18,19], and Hounsfield units (HU) of the solid component of the tumor in unenhanced images.

The dimensional data were acquired by measuring the maximum diameter of the tumor in the axial plane in the postcontrast images [18,19,20]. Well-defined margins were characterized by tumor circumference with a “pencil-thin”, sharp appearance >90% in postcontrast images (including contact with renal parenchyma, collecting system, and adjacent adipose tissue) [18,19,20]. To acquire these data, a window with width and level values of W: 400 L: 50, respectively, was used [18,19,20]. When the tumor comprised ≥50% of one or more well-delimited cystic spaces characterized by fluid attenuation values (i.e., ≤20 HU), the tumor was defined as cystic, while if the cystic component was absent or <50% of the entire ccRCC volume, the tumor was considered solid [18,19,20]. Tumor necrosis, evaluated during the nephrographic or excretory phases, is characterized by the presence of hypodense areas that do not show contrast enhancement or evident and delineated walls [18,19,20]. These imaging features distinguish tumor necrosis from the cystic component [18,19,20]. Calcifications are defined as high-density spots or plaques. Doubtful cases of calcifications were evaluated considering maximum HU values greater than 60 HU [18,19,20]. The presence of areas with HU blood values (i.e., +30 to +80) characterized intralesional hemorrhage [18,19,20]. When the calcifications and areas of tumoral hemorrhage presented similar HU values, the distinction was made through the evaluation of the morphology by two radiologist experts in oncological imaging (G.F., 9 years of experience; C.A.M., 13 years of experience) [18,19,20]. Tumor infiltration, evaluated in the postcontrast phases, was characterized by the development and growth of tumor tissue into the surrounding healthy tissue [18,19,20]. Hydronephrosis, assessed on postcontrast images, was characterized by dilation of the urinary tracts [17,18,19]. Thrombosis of the renal artery or vein was evaluated on postcontrast images and was defined as an intraluminal filling defect of thrombotic nature [18,19,20]. Collecting system invasion, evaluated in postcontrast images in the excretory phase, was defined as collecting system endoluminal filling defects by tumor tissue [18,19,20].

### 2.3. Lipid Metabolism Imaging Features

Evaluation of imaging features expressing ccRCC lipid metabolism was performed by measurement of tumoral HU and by quantification of abdominal adipose tissue compartments. Tumoral HU was performed on unenhanced images by placing a region of interest (ROI) in the solid component of the tumor and acquiring the mean of the HU present within the ROI. These data allowed us to have an estimate of the quantity of lipids present inside the tumor cells. Adipose tissue has HU values from −50 to −100; therefore, the lower the HU values present in correspondence with the solid component of the tumor, the greater the intracellular accumulation of lipids.

Total abdominal tissue (TAT), visceral adipose tissue (VAT), and subcutaneous adipose tissue (SAT) were measured using a semi-automatic function of Horos v.4.0.0 RC2 software, which allowed us to analyze all cross-sectional CT images by selecting the typical HU values of the adipose tissue. The data were obtained as areas (cm^2^) on a single axial image located 3 cm above the lower edge of L3, as previously described [21].

All the ROIs were performed by a consensus of 2 radiologists (G.F. and C.A.M., with 9 years and 13 years of experience, respectively) who were blinded to the clinical data.

### 2.4. Statistical Methods

Three sets of analyses were performed. First, we tabulated demographics as well as clinical–pathological and CT-related tumor features according to ADFP expression (yes vs. no). Second, quantification of abdominal adipose tissue compartments, namely, VAT, SAT, and TAT, were compared according to ADFP expression and Fuhrman tumor grade (low-grade [G1–2] vs. high-grade [G3–4]). Finally, tumoral HU were also compared according to ADFP expression and Fuhrman tumor grade. For all comparisons, descriptive statistics included frequencies and proportions for categorical variables, while medians and interquartile ranges (IQRs) were reported for continuously coded variables. The Wilcoxon rank sum test, Pearson’s Chi-square test, and Fisher’s exact test were used to examine the statistical significance of differences in medians and proportions, respectively, between groups. All tests were two-sided, with a level of significance set at *p* < 0.05. The R software environment for statistical computing and graphics (version 4.1.2, R foundation for Statistical Computing, Vienna, Austria) was used for all analyses.

## 3. Results

Overall, 197 patients were identified (Table 1). Of these, 45 (22.8%) had ADFP expression.

Among CT-related tumor features, patients with ADFP expression more frequently had hydronephrosis (8.9% vs. 1.3%, *p* = 0.025). The distribution of other CT-related findings between groups is also shown in Table 1.

Among demographics and clinical-pathological tumor features, not clinically, as well as statistically significant differences emerged between patient groups.

**Figure 1:**
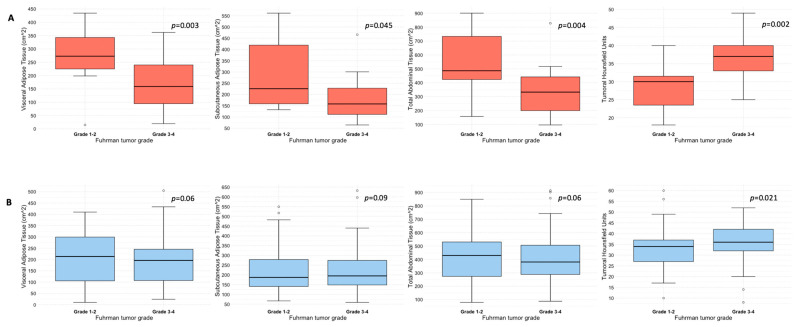
Box and whisker plots depicting adipose tissue compartments measurement and tumoral HU according to ADFP expression ((**A**), yes vs. (**B**), no) and Fuhrman tumor grade.

Quantification of abdominal adipose tissue compartments in patients with and without ADFP expression, stratified according to Fuhrman tumor grade, is shown in Table 2. Specifically, patients with ADFP expression and low-grade tumors (*n* = 13) exhibited higher median values of either VAT (273 vs. 159 cm^2^, *p* = 0.03), SAT (226 vs. 158 cm^2^, *p* = 0.045), or TAT (486 vs. 333 cm^2^, *p* = 0.004) compared with their high-grade counterparts (*n* = 32). Conversely, no statistically significant differences emerged between patients with low-grade (*n* = 66) and high-grade (*n* = 86) tumors without ADFP expression for VAT (213 vs. 196 cm^2^, *p* = 0.6), SAT (187 vs. 195 cm^2^, *p* = 0.9) or TAT (430 vs. 381 cm^2^, *p* = 0.6) (see also Figure 1). Median tumoral HU was higher in patients with high-grade tumors compared to their low-grade counterparts, independently by the presence of ADFP expression: 37 vs. 30 (*p* = 0.002) in patients with ADFP expression and 36 vs. 34 (*p* = 0.021) in patients without ADFP expression (Table 2 and Figure 1). Analysis of abdominal adipose tissue compartments and tumoral HU between low-grade ccRCC patients with and without ADFP expression revealed a higher quantity of VAT in ccRCC patients with ADFP expression, meeting the threshold value (273 vs. 213 cm^2^, *p* = 0.05), while no statistically significant differences were found for SAT (226 vs. 187 cm^2^, *p* = 0.2), TAT (486 vs. 430 cm^2^, *p* = 0.075), or tumoral HU (30 vs. 34, *p* = 0.07). Finally, analysis of abdominal adipose tissue compartments and tumoral HU between high-grade ccRCC patients with and without ADFP expression showed no statistically significant differences for VAT (159 vs. 196 cm^2^, *p* = 0.3), SAT (158 vs. 195 cm^2^, *p* = 0.06), TAT (333 vs. 381 cm^2^, *p* = 0.11), or tumoral HU (37 vs. 36, *p* = 0.9).

## 4. Discussion

**Figure 2:**
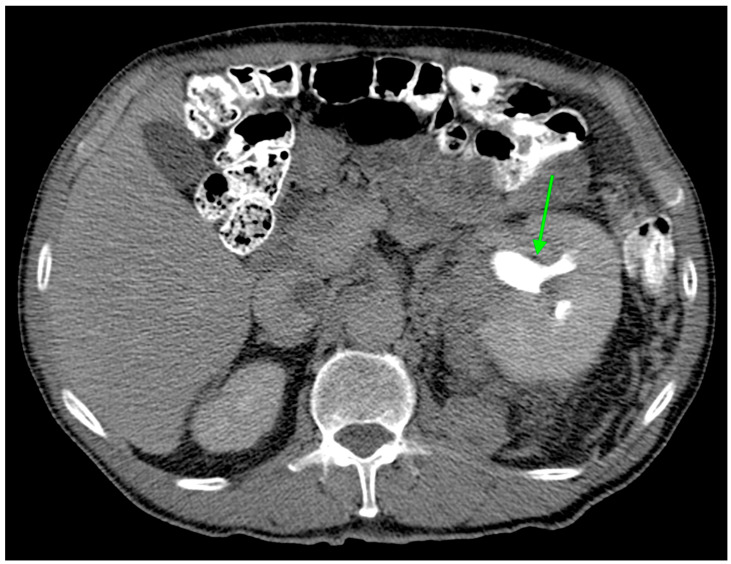
Axial CT image during excretory phase showing ccRCC with AFDP gene expression with hydronephrosis (green arrow).

**Figure 3:**
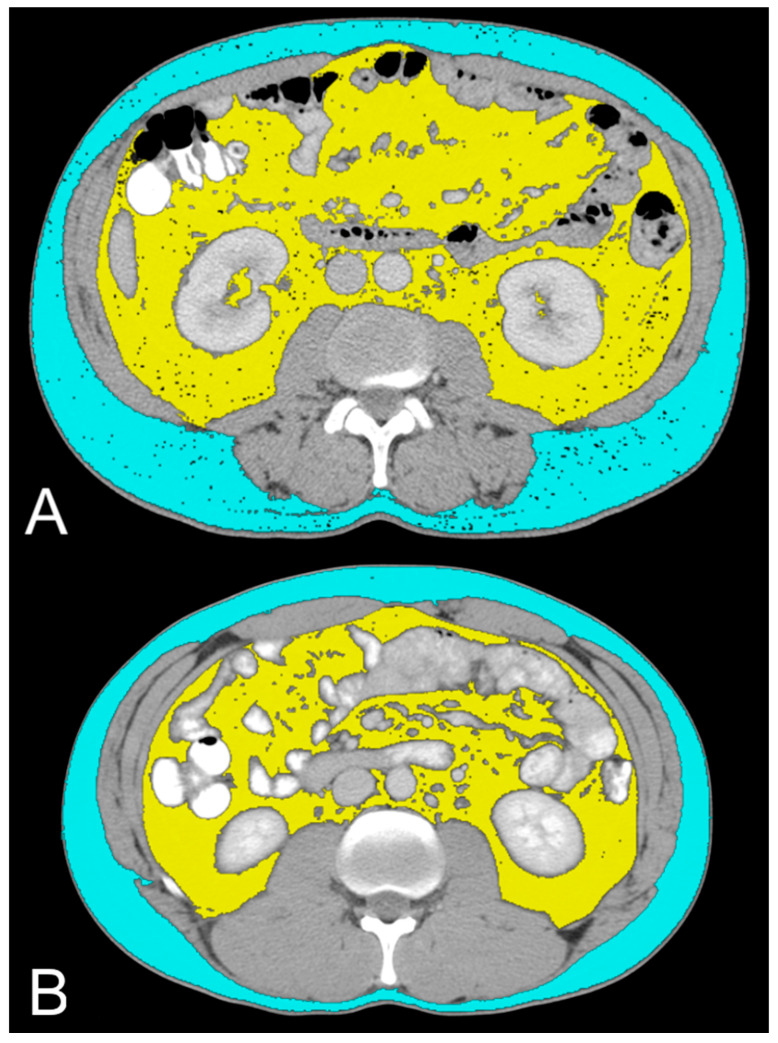
Axial CT images showing VAT segmented in yellow and SAT segmented in light blue in low-grade ccRCC patients with ADFP expression (**A**) and high-Fuhrman-grade ccRCC patients with ADFP expression (**B**).

**Figure 4:**
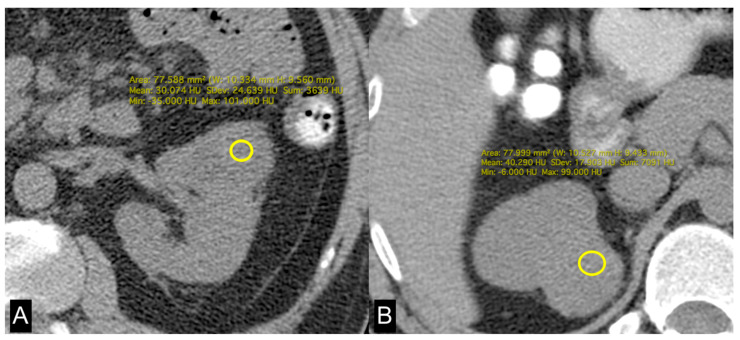
Unenhanced axial CT images of patients with low-grade ccRCC with ADFP expression (**A**) and high-grade ccRCC with ADFP expression. (**B**) Yellow ROIs with different mean attenuation values of tumoral HU (HU: 30 and 40,2, respectively).

In this study, we evaluated the CT features of ADFP expression in ccRCC patients. A significant correlation was found with hydronephrosis (*p* = 0.025) (Figure 2). Furthermore, greater quantities of TAT, VAT, and SAT were found in low-Fuhrman-grade ccRCC patients with ADFP expression compared to high-Fuhrman-grade ccRCC patients with ADFP expression (*p* = 0.004, *p* = 0.003 and *p* = 0.045, respectively) (Figure 3). Significantly lower tumoral HU values were found in low-Fuhrman-grade ccRCC patients with ADFP expression compared to high-Fuhrman-grade ccRCC patients with ADFP expression (*p* = 0.002) (Figure 4), and significantly lower values of tumoral HU in low-Fuhrman-grade ccRCC patients without ADFP expression were found compared to high-Fuhrman-grade ccRCC patients without ADFP expression (*p* = 0.021).

Hydronephrosis was found to be a radiogenomic feature of ccRCC with ADFP expression (Figure 2). This link could be due to an indirect association. For instance, ADFP’s role in lipid metabolism might influence tumor characteristics such as size, invasiveness, or vascularity, which in turn could contribute to the development of hydronephrosis due to tumor obstruction, making hydronephrosis a typical feature of ccRCC with ADFP expression.

It is currently understood that surplus adipose tissue, especially VAT, plays an active role in the pathogenesis of RCC. Specifically, an elevated presence of VAT has been observed in ccRCC patients [22]. The excessive adipose tissue prompts the release of HIF-1 due to insufficient oxygen supply to the adipocytes, alongside irregular secretion of adipokines like leptin, adiponectin, resistin, and visfatin. This mechanism may potentially establish a connection between obesity and the development of RCC [23,24,25].

In ccRCC, the link between ADFP and HIF is of particular interest due to their involvement in the tumor microenvironment and metabolic dysregulation characteristic of ccRCC. ADFP is upregulated in ccRCC via a HIF-2α dependent mechanism [26]. It has been demonstrated that the lipid phenotype in tumor cell lines shows elevated ADFP expression in ccRCC, specifically dependent on HIF-2α [27]. HIF-1α is expressed in both mature adipocytes and progenitor cells, whereas HIF-2α is expressed in differentiated adipocytes but not in preadipocytes, indicating a specific role for HIF2α in mature adipocytes [28,29]. Studies using animal models have demonstrated that HIF-1α protein levels [30] and HIF DNA-binding activities [31] are elevated in the adipose tissue of obese mice. Additionally, HIF-2α protein levels are increased in the adipose tissue of mice fed a high-fat diet for 4 weeks [32]. HIF causes accumulation of lipid droplets by repressing the expression of carnitine palmitoyltransferase 1A (CPT1A), a rate-limiting enzyme that regulates the transport of fatty acids into the mitochondria, thereby promoting fatty acid beta-oxidation [26]. CPT1A expression is repressed by both HIF-1α and HIF-2α, which, through their activity, interfere with fatty acid transport into the mitochondria and determine the accumulation of fatty acids and lipid droplets [26].

Research has shown that ccRCC tumors often exhibit dysregulated lipid metabolism and are associated with increased expression of ADFP [9]. In the context of ccRCC, ADFP expression may contribute to the accumulation of lipid droplets within tumor cells, which is a common feature of this histotype [9]. Indeed, ccRCC is known for its elevated lipid content, including cholesterol, cholesterol esters, and phospholipids in the cytoplasm, resulting in a distinct “yellow” appearance upon gross examination and primarily consisting of “clear cells” under routine hematoxylin–eosin staining [33,34].

Yao et al. found that low-grade ccRCCs tend to exhibit higher expression levels ADFP, which plays a role in fatty acid uptake and the formation of lipid droplets within cells, compared to high-grade tumors The overexpression of ADFP is associated with increased intracellular lipid storage in low-grade ccRCCs relative to high-grade tumors [9,35]. Choi et al. demonstrated that the decreased attenuation detected in low-grade tumors on unenhanced CT images is likely indicative of a higher lipid content within these tumors [36].

Consistent with these findings, we observed higher abundances of TAT, VAT, and SAT in low-Fuhrman-grade ccRCC patients expressing ADFP compared to high-Fuhrman-grade ccRCC patients with ADFP expression. Additionally, we noted significantly lower tumoral HU values in low-Fuhrman-grade ccRCC patients expressing ADFP compared to high-Fuhrman-grade ccRCC patients expressing ADFP (*p* = 0.002), as well as significantly lower HU values in low-Fuhrman-grade ccRCC patients without ADFP expression compared to high-Fuhrman-grade ccRCC patients without ADFP expression (*p* = 0.021). This explains how the excessive amount of adipose tissue determines an increase in HIF with associated greater expression of ADFP and a consequent increase in intracellular lipid droplets; the relationship between HIF and ADFP could be further strengthened by VHL gene mutation, which inactivates HIF degradation. 

Backing these findings, the comparative examination of low-Fuhrman-grade ccRCC patients with and without ADFP expression revealed a higher quantity of VAT in ccRCC patients with ADFP expression, meeting the threshold value (*p* = 0.005), along with a trend toward significance indicating elevated TAT values and decreased tumoral HU values in ccRCC patients with ADFP expression (*p* = 0.075 and *p* = 0.070, respectively). However, the study acknowledges several limitations, including the small number of patients included and the presence of confounding factors related to the expression of other genes associated with the accumulation of lipid droplets in ccRCC, such as ancient ubiquitous protein 1 (AUP1) and acyl-CoA synthetase 3 (ASCL3) [37,38]. To further elucidate the role of ADFP in ccRCC, future studies with larger sample sizes and without the presence of confounding gene expressions involved in intracellular lipid droplet accumulation are warranted. A further limitation of the study is the small sample size; the study included only 197 patients, which may limit the generalizability of the findings. A larger sample size would provide more robust statistical power and enhance the reliability of the results. The retrospective design could introduce selection bias and limit the ability to establish causal relationships between ADFP expression and imaging features. Regarding the imaging technique, while CT imaging was utilized for radiogenomic analysis, the study did not mention the potential variability in imaging protocols, equipment, and interpretation among different institutions. Standardization of imaging techniques could enhance the reliability and reproducibility of the results. Biological variability among patients, such as variations in tumor biology, metabolism, and host factors, could confound the observed associations between ADFP expression, imaging features, and clinical outcomes. There was also incomplete data; the study did not provide information on certain variables that could influence the relationship between ADFP expression and imaging features, such as patient comorbidities, medications, or lifestyle factors. Lastly, there may be a risk of publication bias, as studies with statistically significant findings are more likely to be published, while those with nonsignificant or negative results may remain unpublished, leading to an overestimation of the observed associations.

## 5. Conclusions

This study explored the correlation between CT imaging features and ADFP expression in ccRCC patients. The findings reveal that ADFP expression is associated with specific radiogenomic features, notably hydronephrosis and higher quantities of TAT, VAT, and SAT in low-grade ccRCC patients. Additionally, lower HU values in low-grade tumors reflect higher lipid content, which is consistent with increased ADFP expression. These results underscore the potential of radiogenomics to provide non-invasive insights into tumor biology and heterogeneity, offering significant implications for personalized treatment strategies.

## Figures and Tables

**Table 1:** Descriptive characteristics of the study population according to ADFP expression.

Characteristic	Overall *n* = 197 ^1^	ADFP Expression		*p*-Value ^2^
		Yes *n* = 45 (22.8%) ^1^	No *n* = 152 (77.2%) ^1^	
***Clinical–pathological features***				
**Age** (years)	59 (51, 69)	58 (53, 68)	60 (50, 69)	0.8
**Sex** (Males)	130 (66.0%)	31 (68.9%)	99 (65.1%)	0.6
**Race/ethnicity** (Caucasian)	182 (91.5%)	42 (93.3%)	140 (92.1%)	0.9
**History of cancer**	35 (17.8%)	9 (20.0%)	26 (17.1%)	0.7
**Primary tumor size** (mm)	53 (38, 81)	54 (45, 76)	53 (37, 83)	0.4
**Laterality** (left)	92 (46.7%)	23 (51.1%)	69 (45.4%)	0.5
**Tumor grade** (Fuhrman)				0.08
Low-grade (G1–2)	79 (40.1%)	13 (28.9%)	66 (43.4%)	
High-grade (G3–4)	118 (59.9%)	32 (71.1%)	86 (56.6%)	
**Tumor stage**				0.5
Stage I	103 (52.3%)	22 (48.9%)	81 (53.3%)	
Stage II	18 (9.1%)	2 (4.4%)	16 (10.5%)	
Stage III	49 (24.9%)	14 (31.1%)	35 (23.0%)	
Stage IV	27 (13.7%)	7 (15.6%)	20 (13.2%)	
***CT-based features***				
**Collateral vascular supply**	111 (57.5%)	28 (63.6%)	83 (55.3%)	0.3
Tumor margins				0.5
Ill defined	69 (35.0%)	14 (31.1%)	55 (36.2%)	
Well-defined	128 (65.0%)	31 (68.9%)	97 (63.8%)	
**Tumor composition**				0.5
Solid	183 (92.9%)	41 (91.1%)	142 (93.4%)	
Cystic	14 (7.1)	4 (8.9%)	10 (6.6%)	
**Tumor necrosis**				0.13
0%	12 (6.0%)	5 (11.1%)	7 (4.6%)	
1–33%	117 (59.4%)	29 (64.4%)	88 (57.9%)	
34–66%	47 (23.9%)	6 (13.3%)	41 (27.0%)	
>66%	21 (10.7%)	5 (11.2%)	16 (10.5%)	
**Tumor growth pattern**				0.3
Endophytic	13 (6.6%)	1 (2.2%)	12 (7.9%)	
Exophytic < 50%	59 (29.9%)	17 (37.8%)	92 (60.5%)	
Exophytic ≥ 50%	125 (63.5%)	27 (60.0%)	48 (31.6%)	
**Calcifications**	39 (19.9%)	10 (22.2%)	29 (19.2%)	0.7
**Signs of infiltration**	5 (2.5%)	2 (4.4%)	3 (2.1%)	0.3
**Hydronephrosis**	6 (3.0%)	4 (8.9%)	2 (1.3%)	**0.025**
**Thrombosis or infiltration of renal artery**	4 (2.0%)	0 (0%)	4 (2.6%)	0.6
**Thrombosis or infiltration of renal vein**	15 (7.6%)	3 (6.6%)	12 (7.8%)	0.9
**Collecting system invasion**	61 (31.0%)	15 (33.3%)	46 (30.3%)	0.7
**Perinephric fat stranding**	103 (53.9%)	24 (55.8%)	79 (53.4%)	0.8
**Gerota’s fascia thickening**	74 (37.6%)	13 (29.5%)	61 (41.5%)	0.2
**Intralesional hemorrhage**	5 (2.5%)	1 (2.2%)	4 (2.6%)	0.9

^1^ Median (IQR); *n* (%) ^2^ Wilcoxon rank sum test; Fisher’s exact test; Pearson’s Chi-square test. Abbreviations: ADFP, adipose differentiation-related protein. Values in bold indicate statistical significance set at *p* < 0.05.

**Table 2:** Adipose tissue compartments measurement and tumoral Hounsfield units according to ADFP expression and Fuhrman tumor grade.

***No ADFP Expression*** **(*n* = 152)**			
	**Low-grade (G1–2)** ***n* = 66 (43.4%) ^1^**	**High-grade (G3–4)** ***n* = 86 (56.6%) ^1^**	***p*** **-value ^2^**
**VAT** (cm^2^)	213 (105, 299)	196 (107, 245)	0.6
**SAT** (cm^2^)	187 (141, 278)	195 (148, 274)	0.9
**TAT** (cm^2^)	430 (274, 530)	381 (288, 506)	0.6
**HU**	34 (27, 37)	36 (32, 42)	**0.021**
***ADFP Expression*** **(*n* = 45)**			
	**Low-grade (G1–2)** ***n* = 13 (29%) ^1^**	**High-grade (G3–4)** ***n* = 32 (71%) ^1^**	***p*** **-value ^2^**
**VAT** (cm^2^)	273 (225, 343)	159 (95, 240)	**0.003**
**SAT** (cm^2^)	226 (159, 419)	158 (112, 228)	**0.045**
**TAT** (cm^2^)	486 (423, 733)	333 (200, 442)	**0.004**
**HU**	30 (24, 32)	37 (33, 40)	**0.002**

^1^ Median (IQR); *n* (%) ^2^ Wilcoxon rank sum exact test; Wilcoxon rank sum test; Fisher’s exact test. Abbreviations: ADFP, adipose differentiation-related protein; VAT, visceral adipose tissue; SAT, subcutaneous adipose tissue; TAT, total adipose tissue; HU, Hounsfield units. Values in bold indicates statistical significance set at *p* < 0.05.

## Data Availability

The data presented in this study are openly available in The Cancer Imaging Archive (https://www.cancerimagingarchive.net/collection/tcga-kirc/ accessed on 1 November 2019).
